# “We Want Good Education for All of Us” – A Participatory Quality Improvement Approach

**DOI:** 10.3389/fmed.2022.538398

**Published:** 2022-03-22

**Authors:** Friederike Holderried, Christine Krejci, Martin Holderried, Maria Lammerding-Koeppel, Teresa Loda, Stephan Zipfel, Anne Herrmann-Werner

**Affiliations:** ^1^Department of Gastroenterology, Hepatology and Infectious Diseases, Internal Medicine, University Hospital Tübingen, Tübingen, Germany; ^2^Faculty V of Mechanical Engineering and Transport Systems, Technical University of Berlin, Berlin, Germany; ^3^Department of Medical Structure, Process and Quality Management, University Hospital Tübingen, Tübingen, Germany; ^4^Competence Centre for University Teaching in Medicine Baden-Württemberg, Faculty of Medicine, University of Tübingen, Tübingen, Germany; ^5^Tübingen Institute for Medical Education, University of Tübingen, Tübingen, Germany; ^6^Deanery of Students’ Affairs, Faculty of Medicine, University Hospital Tübingen, Tübingen, Germany; ^7^Department of Psychosomatic Medicine and Psychotherapy, Internal Medicine, University Hospital Tübingen, Tübingen, Germany

**Keywords:** Communities of Practice, quality improvement, quality management, medical students, identification

## Abstract

**Introduction:**

In ever changing conditions, medical faculties must face the challenge of preparing their medical students as best as possible for the demands of their future work. This requires involving all stakeholders, especially medical students in the constant redefinition of medical curricula. Using the idea of “Communities of Practice” as conceptual framework, this study looks at semester spokespeople as an example for participatory quality management.

**Methods:**

We conducted focus-group interviews with semester spokespeople at a German Medical Faculty. Data was recorded, transcribed, and analysed using MAXQDA. The interviews were analysed using meaning condensation method.

**Results:**

Eleven out of 48 semester spokespeople took part. We found seven topics that fell within three main categories: (1) role of the semester spokesperson, (2) role of the fixed meeting, and (3) contact and commitment. Communities of Practice principles could be aligned to topics and categories.

**Discussion:**

The idea of semester spokespeople based on the concept of Communities of Practice are useful in the quality management processes of a medical school and lead to greater involvement of medical students, identifying their needs. The reciprocal commitment among all stakeholders fosters mutual understanding and collaboration. Future studies could investigate the underlying motivational factors of dedicated students and how to transfer these characteristics to a larger cohort.

## Introduction

Medical faculties do not only teach their students for the here and now, but also should consider the future challenges doctors will face. This means that the content and structure of teaching should already be anticipating how everyday medical practice will develop over the next 10 years (1, 2). This challenge is not new, but becomes more and more evident in times of major changes affecting medicine and healthcare, such as increasing digitalisation, the scarcity of resources, and the redefinition of the physician’s role in the inter-professional healthcare team (3). Thus, upcoming challenges should be actively embraced, and any new competences required by students should be integrated into the curriculum without delay.

Medical universities must constantly re-define their curriculum and successfully carry out continuous change processes in accordance with external influences to meet these requirements for the long-term. In many places, such change processes are perceived to be difficult and are met with little understanding and willingness among individual participants (4, 5). The well-established methods are perceived as safe and simple, while the restructuring measures are deemed to be tedious and unnecessary (6).

To counteract these difficulties from the outset and prevent any associated deterioration in the quality of teaching, all stakeholders should be involved in the continuous improvement processes. The stakeholders’ knowledge and opinions should be listened to and integrated into these processes (7). Only then can a curriculum remain flexible enough to constantly adapt to rapidly changing requirements and easily adopt and implement necessary changes or innovations (8).

In this study, such a concept of integration was presented, used, and evaluated after a 10-year period. It explicitly looks at the participation of students in the change processes (7) as well as to direct quality assurance regarding organisational and content issues of the curriculum (7, 9).

Ten years ago, the curriculum for undergraduate medical education at the University of Tübingen underwent profound reform. One aspect of the reform was the focus of practical and communication skills alongside traditional medical education. Furthermore, the concept of semester spokesperson was introduced during this reform meaning medical students from each semester voted for representatives. These representatives were included in the faculty’s steering and decision processes *via* a direct exchange with the deanery embedded in a holistic organisational development concept [Communities of Practice (CoP)] (3, 10, 11). CoPs have been used for decades by large organisations in order to bring together the existing knowledge, ideas, and motivation of employees in the company and to integrate them into any improvement processes (10). They consist of groups of employees with common problems or ideas who want to share prior knowledge as well as any new ideas with each other, and who therefore meet regularly on a voluntary basis (11, 12).

This idea is premised on the theory that individual employees with common problems or ideas are already forming informal groups without external influence to exchange knowledge, tackle problem areas, and facilitate processes. To make use of the knowledge and skills of these groups, environmental factors can be implemented that advance the work and preservation of these groups. To this end, Wenger et al. describes seven beneficial principles (11) (detailed information about CoPs and the seven beneficial principles are shown in Table 1).

**TABLE 1:** Comparison of the seven principles of Communities of Practice with the participatory quality management at Medical Faculty Tübingen.

Seven principles*		
	Communities of Practice (CoP)	Quality management concept Tübingen: Structure, rights, and responsibilities
1. Design for evolution	The CoPs are usually • Formed **based on pre-existing personal networks** • **Voluntarily** participated in • Created from supporting organisational and structural **framework conditions** (communication structures, problem-solving meetings) to facilitate exchange within the CoP	The formation of a CoP among students is initiated by an **internal election** of officially recognised semester spokespeople (4 per semester cohort) on a **voluntary basis**, who are **invested with rights and responsibilities**
2. Open dialogue between inside and outside perspectives	Group members know • What information is relevant to the process, • Which stakeholders are involved in the event, and • Which proposed solutions have already been raised in the group Outsiders provide • Offer new perspectives on incidents or problems and • Contribute to a solution It is important to **create structures that enable dialogue** not only **within the group but also with outsiders**	The faculty support the semester spokespeople by **introducing a communication structure consisting of:** regular fixed meetings during the semester (3 per semester), with the **faculty representative (Vice Dean for Teaching, Dean’s office management), representatives of the main departments, and the semester spokespeople participating,** giving the spokespeople the opportunity to report the **concerns and problems** of their fellow students that have been gathered over the semester and to **seek solutions together** with all relevant representative bodies. The spokespeople can then pass these solutions back to their fellow students
3. Invite different levels of participation (see Figure 1)	Extent of participation in the CoPs vary: **“Core Group”** • Actively participates in all discussions and tackles the upcoming projects • Heart of the CoP and constitutes just 10–15% of the group **“Active Group”** • Participates in discussions from time-to-time and takes part in individual projects that interest them • Group size approximately 15–20% **“Peripheral group”** • Are rarely involved in discussions and acts more as observers • But, can also contribute knowledge, depending on the subject area There are no fixed boundaries between the groups and many participants change groups depending on their level of interest **“Outsiders”** • Non-group members • Compulsory participation is not useful It is the task of the Core Group of the **Community Coordinators** to **build bridges**, by setting up discussions and ensuring the flow of information	Members of the “**Core Group”** of the CoP **contact their fellow students** about issues regarding study-related organisation and quality control (CoP principle 3). This contact allows the early detection of possible problems or moods within a semester cohort **(insider perspective)** The spokespeople recruit semester colleagues (**“Active group”**) for special tasks to support them with gathering information (concerning current issues) and developing a work force (semester activities) The spokespeople maintain a list of all semester colleagues, so they can reach them in case they need to gather or distribute information
4. Develop private and public community spaces	Provide **public events** involving all group members to obtain active **cooperation**. This is primarily about building new connections and reinforcing old connections. Moreover, it also encourages **private exchanges** between the group members. In this safe space, **problem areas and personal views** can be discussed without fear of repercussions	**Regular fixed meetings** during the semester (3 per semester) Monthly private meetings of all spokespeople of one semester to discuss current issues
5. Focus on value	The popularity of the CoP is a result of the fact that the **organisations and participants gain clear benefits** from the activities. At the same time, this added value is initially aimed at concrete projects, but a **systematic body of knowledge** that is perceived and valued among the members gradually develops	Furthermore, the semester spokespeople can **revert to the experience of older semester spokespeople** through the cross-semester event
6. Combine familiarity and excitement	Successful CoPs offer their members **repetitive structures** that, on the one hand create a **feeling of familiarity,** and on the other offer new events to mobilise potential members	The core group, with support from fellow students in the active group, **organises events that promote cooperation** (semester parties, joint book orders, etc.)
7. Create a rhythm for the community	The speed and vitality of a CoP is decisively determined by the **frequency and nature of the joint meetings**	**Regular fixed meetings** during the semester (3 per semester)

*(11).

**FIGURE 1:**
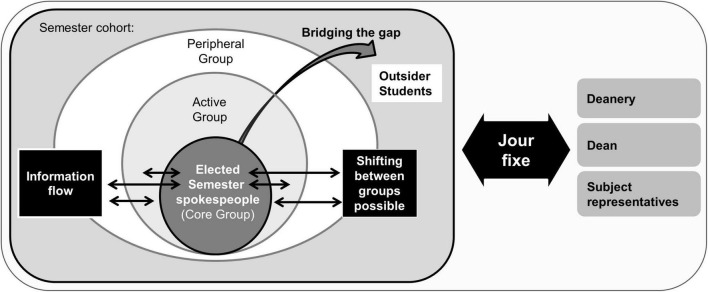
CoP: Communication structure and group affiliation (11).

At the Medical Faculty Tübingen, one part of the quality management was developed and implemented following this concept, as it has shown to be suitable for an educational context (13). At an introductory party weekend for freshmen at the beginning of each semester, students can volunteer to be a semester spokesperson. All other students have to give their approval and from then on, the thus elected semester spokespeople (“core group” consisting of four members per semester) function as information gatherers and are responsible for the information flow between the entire semester cohort (“peripheral group”) and the faculty. To simplify this task, a communication structure of three regularly fixed meetings per semester was implemented (“Jour Fixe”). At these meetings, all stakeholders (spokespeople, dean, subject representatives, and deanery) of the curriculum came together to discuss current issues. If necessary, the spokespeople could recruit semester colleagues for special tasks concerning information gathering or workforce tasks (e.g., for semester activities) as shown in Figure 1.

The aim of this study is to examine, 10 years after its introduction, how students understand their roles, whether they were adequately supported by the faculty, and how they experienced the introduction of the CoP.

## Materials and Methods

To date, only a few studies have looked at the extent to which students can be actively involved in quality assurance and the positive effects as well as challenges that implementing this involvement poses for the students. Therefore, we selected a qualitative research approach. Focus group interviews allow for comprehensive insight into the experiences and views of the participants (14, 15). The recommendations for the documentation of qualitative data were compiled during the process of conducting the study (16). To ensure consistency, interview guidelines were created. These included three blocks of open questions, which served as discussion triggers.

### Setting

The study was conducted at the Medical Faculty of Tübingen University with approximately 2,400 students studying human medicine.

### Participants and Process of the Interviews

All 48 semester spokespeople of the Tübingen Medical Faculty were invited by email to carry out the focus group interview, and eleven semester spokespeople voluntarily took part in the study. Informed consent was given orally. Eight participants were male, three were female. The participants represented students from semester 3 to 9 and were in office on average 3.7 semesters (SD ± 1.2). All participants were divided randomly into two groups with five and six spokespeople in the groups. Both interviews took place in two fixed meetings lasting 115 min within a short timeframe, one after the other. As a result, the likelihood of acute external events or exchanges between participants of the two groups influencing the other was kept as low as possible. The interviews were moderated by one person (FH), who has additional professional qualifications in the field of communication and team supervision. The following three questions were posed (S1 and S2 file):

1. How do you see your role as a semester spokesperson?
2. How would you describe the role of the Jour Fixe?
3. How would you describe the interpersonal implications coming along with being a semester spokesperson?

### Data Collection and Analysis

All data were pseudonymised. The focus group interviews were digitally recorded with the prior consent of all participants. There was a 2-h time frame for the interview which lasted 115 min each. These sessions were transcribed, anonymised, and transferred to MAXQDA Version X. At the beginning, the researchers FH, CK, and AHW read the transcripts to get a general sense of the documents. The text transcribed from the interviews was analysed using systematic text condensation, an inductive approach by Malterud (17). First, the material was read to obtain an overall impression and noting tentative themes. Then, the units of meaning were identified and several aspects of the focus groups were coded (see also Appendix Table 1 for an overview of the codes). In the next step, the meaning was condensed within the codes. Finally, the contents were synthesised in order to generalise descriptions and concepts of the main themes. This coding process was conducted separately by FH, CK, and AH-W. Subsequently, the results were collated and the resulting coding systems were combined in a discursive process. In the next step, the meaningful units were grouped together into more comprehensive categories and sub-categories (topics) in an iterative process. We tried to develop precise categories and topics that allow distinction. Any disagreements between the researchers were resolved by consensus in group discussion. The saturation has been reached when no theoretically relevant similarities and differences can be discovered in the data material (18–21). It turned out that the last interview did not address any new topics regarding our research question. This process was repeated until all the authors involved saw the interview texts represented in the topics and categories set out below. The meaning of the categories and codings were transferred into descriptive statements underlined with relevant quotes.

### Ethical Approval

The study received ethical approval from the Ethics Committee of Tübingen Medical Faculty (No. 058/2011A).

## Results

### Main Categories and Topics

The evaluation of the focus group interviews gave rise to seven topics in three main categories (see Table 2).

**TABLE 2:** Categories and topics.

Main category	Topics
1. Role of the semester spokesperson	The semester spokespeople must gather and prioritise the queries of their fellow students The semester spokespeople must set clear limits on the extent to which they can support their fellow students
2. Role of the fixed meeting	The fixed meetings promote transparency The fixed meeting shows structural limits
3. Contact and commitment	The fixed meeting offers the semester spokespeople contacts and support The “face-to-face” meetings facilitate the connection between the Dean’s office and the semester spokespeople The continuous connection of all involved parties encourages community

#### Main Category 1: Role of Spokesperson

When introducing the position of “semester spokesperson,” a job description with rights and responsibilities was defined through cooperation between the deanery and students (see Table 1). This included both the content-related/structural tasks as well as the social duties of the semester spokesperson. Two topics arose regarding purpose of this role.

##### Topic 1: Information Gathering and Prioritisation

When they take on their role, semester spokespeople are faced with the challenge of being clear about their role and the associated responsibilities.

*“[*…*] also if I wrote an email using the mailing list, I would think: ‘Wow, now I’m writing an email to 200 people at once”’ (Student A, 3rd semester, 3rd semester in office).*

The pooling of information from the semester plays an important role. The semester spokespeople received a lot of feedback from their fellow students prior to the regularly scheduled fixed meetings.

*“So I saw myself as being like a sewer system in order to pool the streams that are continually emerging and then, together with the people, discuss the problems with the relevant contact within the faculty” (Student B, 8th semester, 3rd semester in office).*

Also, the prioritisation of individual topics presented the students with a difficult decision, namely how to order the concerns of their fellow students by importance.

*“[*…*] in the very first [fixed meeting] session, I submitted such a long list; I spoke lots and lots and covered an insane amount, and I got a few knocks from the other students for that [*…*]” (Student C, 5th semester, 5th semester in office).*

To reduce the burden of responsibility, decisions are frequently made and communicated by the team of semester spokespeople.

*“We now always make decisions together and there is also a certain degree of security if four of us together say ‘Hey, we want to do it like this or like that [*…*]!”’ (Student D, 6th semester, 6th semester in office).*

##### Topic 2: Setting Limits

As the semester spokespeople are quickly perceived as their fellow students’ first point of contact, they must define the limits of their capacity as their workload increases.

This is not easy for the students, depending on the content and vehemence of the request, and requires a sure understanding of the role.

*“[*…*] then you somehow have to learn how to separate yourself. If fellow students say, ‘but you are the semester spokesperson,’ you have to be self-confident and say ‘No, I’m not the fool in charge of everything”’ (Student E, 5th semester, 5th semester in office).*

This helped to invoke the students’ own value system. Values such as fairness and tolerance toward all parties, objectivity regardless of their own study situation, as well as confidentiality, respect, and the necessary sense of duty were repeatedly mentioned as decision criteria.

*“[*…*] Respect and understanding of the different positions through which this role is perceived means that we’re not just standing as one person, we have the opportunity to represent, perceive, and convey all of the positions to some extent” (Student F, 3rd semester, 3rd semester in office).*

#### Main Category 2: Fixed Meeting

Over the past few years, topics for the fixed meetings have changed. Initially it was mainly organisational issues (for example attendance requirements, room or timetable management, cancelled teaching modules, and availability of teaching materials). However, in its day-to-day operations, semester spokespeople often introduced topics perceived as urgent in the field of study content (e.g., feedback on didactic concepts that were evaluated poorly in the semester group, overall structure of Tübingen’s curriculum), university examinations, or social activities, such as support for festivities.

##### Topic 3: Transparency

Only a few students were concerned with the structure and organisation of the faculty. For most of the students, concepts like the study committee, dean’s office, or faculty council are at most associated with vague ideas, and there is no precise concept of competencies and responsibilities.

“*[*…*] for people who are neither semester spokespeople nor part of the student council, the dean’s office is something like a ‘Black Box”’ (Student G, 8th semester, 3rd semester in office).*

Regular meetings with the faculty gave the semester spokespeople an insight into all the committees involved in teaching, as well as the decision-making processes and decision-making powers of the individual players. This promoted understanding of the process and helps the semester spokespeople in passing on decisions to their fellow students.

*“What I still appreciate is that I simply understood the structures, [*…*] what there is, [*…*] what the they are like here, who needs to deal with whom; and when you look at it, you understand the whole system” (Student F, 3rd department, 3rd semester in office).*

*“It also helps to the extent that the decision-making processes are somewhat easier to understand and you also have good arguments when you go out of the meeting again [*…*]” (Student B, 8th semester, 3rd semester in office).*

##### Topic 4: Structural Limits

As already mentioned, despite the initial intention to limit the fixed meetings to questions regarding study organisation, they are often used to address urgent topics from other categories. However, these often lack the presence of a responsible contact person (e.g., representatives from smaller specialist areas) or the decision-making power is not in the hands of those present.

Even if the students have received a good impression during their time in office of what can and cannot be changed during a fixed meeting, other topics continue to be brought up.

*“I think you get a certain feeling for what will have an effect and what will not, but even with matters that don’t [have an effect] it is important that they are often addressed [*…*]” (Student H, 6th semester, 6th semester in office).*

In this respect, the students specifically use the opportunity to put forward very urgent or emotional topics, if necessary, to relieve themselves of some of the transferred responsibility.

*“We had a topic at the last fixed meeting and we thought for longer than usual about whether to address it at all. Because [we all already knew] the answer would be: ‘hello, this isn’t working!’ [*…*] But when I actually heard the ‘no’ from the deanery, I had an official confirmation” (Student F, 3rd semester, 3rd semester in office).*

On the other hand, the fixed meeting is specifically used as an instrument of communication with all other semester spokespeople who are also present (see also topic 7).

#### Main Category 3: Contact and Commitment

The frequent contact of the groups involved in the quality assurance process leads to an increased commitment both within individual groups and beyond the group boundaries.

##### Topic 5: Contacts and Support Offered by Fixed Meeting

The regularity, as well as the wide range of professional backgrounds from the contact persons, were a great help for students.

The fact that people with decision-making powers (e.g., the Vice Dean for Teaching) participate in the fixed meetings was considered very helpful. Through direct feedback on their questions, the semester spokespeople received support in the performance of their duties.

*“[*…*] with the decision-makers from the Dean’s office being here, it implies that decisions are really coming directly from the top, and if you receive a confirmation, then this really means something, [*…*] I find it very good for the justification of my role; yes, it bolsters my authority” (Student G, 8th semester, 3rd semester in office).*

Moreover, due to the regular contact and shared tasks, the students lose any reservations in dealing with upper management. This facilitates the accomplishment of shared tasks.

*“[The fixed meeting] somehow slightly lessens the hierarchy, you all just sit at the same table [*…*]” (Student G, 8th semester, 3rd semester in office).*

##### Topic 6: Connection Facilitated by “Face-to-Face” Meetings

Also, the constant connection between the students and the same group of people representing the faculty facilitates collaborative work.

*“[*…*] that you recognise the faces at the fixed meeting and you know who your contact is was really something else, whether I saw the picture on the homepage and knew that our Dean is called xy, or whether he is standing in front of you at the fixed meeting saying ‘come to me with any questions”’ (Student I, 9th semester, 4th semester in office).*

Thus, a feeling of appreciation can be fostered through respectful and appreciative behaviour.

Mutual learning and appreciation make it easier for the semester spokespeople to understand the views of the other group members. Restrictions affecting the group from outside can be recognised and accepted as such. This promotes the students’ problem-solving skills.

*“The fixed meetings contribute to your understanding of the requirements and constraints of the Dean’s office, and even meant that you got to know the people a bit better, and therefore it promotes mutual understanding and I believe it also promotes problem solving skills” (Student G, 8th semester, 3rd semester in office).*

##### Topic 7: Community Encouraged by Continuous Connection

In addition to the connection to the dean’s office, there is also a connection between the semester spokespeople of different semesters. They have the opportunity to learn from each other and to create a sort of institutional memory of a positive experience.

*“[The fixed meeting] promotes exchanges between the semester spokespeople, so that you then know what you might be faced with in the next semester [*…*] I find that type of communication incredibly important” (Student G, 8th semester, 3rd semester in office).*

The students also realised that while they may no longer be of help to their own semester cohort, the initiated changes would bring about improvements for future generations. At the same time, they were grateful for the work done by their predecessors. A strong sense of community emerges from this mutual assistance.

*“You also benefit from the fact that other semesters have already done it—it’s a generation thing, I think [*…*] really you can be satisfied overall, because the changes are usually already visible during the following semester so you can then feel personally satisfied” (Student G, 8th semester, 3rd semester in office).*

*“The semesters before us seem to have been pretty good, because at the moment we have very few things to complain about” (Student J, 3rd semester, 3rd semester in office).*

On the other hand, it creates a bond to their “home faculty” at Tübingen University. They see themselves as part of a valuable whole and feel proud of the work done together.

*“You naturally grow through the tasks associated with being a semester spokesperson. Because we are firmly anchored to the role, I would say that we feel a different affiliation to our semester and therefore also to our uni” (Student D, 6th semester, 6th semester in office).*

*“[*…*] I think it’s important that this institution, that outlasts all semesters, also has an interest in listening to the current generation; personally, for me what would be simply great is, if in the end, I can say ‘Hey at Tübingen, teaching is great because this and that was done compared to Uni XY, and then I can have a certain pride in Tübingen”’ (Student B, 8th semester, 3th semester in office).*

### Communities of Practice as a Teaching Quality Assurance

Considering the interview data regarding the underlying conceptual framework of CoP, a further subrange is particularly noticeable. The students quickly realise how essential it is to reach out to the so-called “outsiders,” that is semester members who cannot be found in any form—be it active or passive—in the CoP. Wenger et al. describe this process as “building bridges” (see Figure 1 and Table 1) (11). A main reason for the inclusion of the “outsiders” is the fact that students, who are not familiar with the function of the semester spokespeople, pass them over in the event of a problem.

*“There are always people who do not even inform us that they are in a muddle about something, and you only find out about it because the lecturer sends you an email: ‘Yes hello, I just want to let you know what’s going on under your nose.’ Those are the times that I have the highest blood pressure in my job” (Student G, 8th semester, 3rd semester in office).*

*“So, I think those who pass over someone [in the function of semester spokesperson], are the ones who only have limited involvement in our semester” (Student G, 8th semester, 3rd semester in office).*

The semester spokespeople suggested ways to overcome this challenge: their frequent presence in lectures, as well as being reachable *via* email, and visiting among the different social groups of the semester in order to achieve the greatest possible visibility of their role.

## Discussion

In this study on the evaluation of a participatory quality assurance concept in teaching, three main categories emerged. The interviews showed that within the Tübingen structure of semester spokesperson, the seven principles of CoP are well reflected (for more details, see Table 1).

The first category deals with the introduction of the students to their positions as semester spokespeople. They realise their exposed position in the semester group and rise to this challenge. To fairly meet the defined tasks, the students rely on their own value systems. Additionally, they facilitate their shared responsibility by working together as a team. They together define the boundaries of their actions in order to not be overwhelmed by their duties in terms of time or content. Through this process of “arriving and finding their place,” these students obtain valuable experiences. The recognition of one’s own value system, as well as the necessity of defining the limits of one’s own capacity, are important skills (22). Additionally, the task of being an “advocate” for their fellow students as a semester spokesperson, through listening and actively querying the needs of their fellow students, might also be useful in later professional live.

The second category deals with the newly created communication structure—the “fixed meeting.” The students are very positive about the fact that through the joint, interactive meetings, they get to know and understand the processes and responsible bodies within a faculty. They recognise that greater knowledge about the decision-making process also contributes to a better understanding of the final decision.

The third category deals with the newly formed “inter-professional curriculum team.” The students not only view their own role as important and feel valued in the team, but also experience and understand the tasks and challenges of other team members. This creates mutual appreciation and a strong sense of togetherness. Through this and the possibility of being able to participate actively in the process, a sense of belonging to one’s own faculty emerges together with a feeling of recognition. These influences can have a positive impact on students. Students who see themselves as an important group member can better deal with stress (23), and also have a lower risk of mental illness (24). Knowing how to be part of a functioning team also has a positive impact on their own professional development (25, 26). Furthermore, knowing one’s own role, and having the confidence to be able to fulfil that role, is an important foundation for working and disseminating knowledge within an inter-professional team (27–29).

Communities of practice have been a great enrichment for large companies for decades. In our faculty, the implementation of structures supporting the development of CoP has also shown positive effects mirroring the results derived from the data collection including the three main categories. These changes in the quality assurance process led to a greater involvement of the students. Due to the close connections with teachers as well as committed fellow students that can be formed within the framework of the communication structure, students are highly motivated to cooperate (30). The learning environment is perceived as supportive and aiding their personal and professional development (31). In addition, the semester spokespeople use their role to actively get students involved in events, who would not otherwise be integrated into the semester group, and thus counter any long-term isolation and associated negative effects (32). They unknowingly take on the role of the Community Coordinators as part of CoP (11, 33, 34). In this role, they also support each other and create a common knowledge which is passed on to the next generation (12).

## Limitations

Qualitative data is always context-dependent, which is why transferability of the acquired data to other areas or settings has to be considered carefully (35). This might be amplified in this case as we only looked at one group of stakeholders (semester spokespeople) with one instrument (focus group) at one particular point of time. Furthermore, we used member-checking to achieve credibility by testing the data and material with some medical students (36). However, it gives a first insight into how a concept coming from a different context can be transferred to medical education and with certain adaptions other faculties, which is also demonstrated by the presence of CoPs in different companies (37). It is more difficult to answer the question of whether the topics, which have occurred in the context of student teaching, are transferrable to patient care and whether, for example, engagement with the role of semester spokesperson facilitates the development of understanding the role of a health professional.

Since the cohort of the semester spokespeople is very small across the faculty, only two focus group discussions could be carried out on a voluntary basis (11/48 participants). However, as we did not find any new topics during the second discussion, we assumed that data saturation concerning our research questions had been achieved. Also, the gender distribution within our focus groups did not reflect the higher female proportion of the general student population, which might have led to a bias in results. However, as it mirrored gender distribution in the group of semester spokespeople, we still consider the results a representative view of our target group. Furthermore, we did not receive any information how the stakeholders perceived the quality or good education which should be investigated in future research.

## Conclusion

This study aims to investigate, after 10 years of the introduction of CoP, what conclusion can be made on how students understand their roles, whether they were adequately supported by the faculty, and how they experienced the introduction of the CoP. Based on the results of the three main category role of semester spokesperson, role of the fixed meeting and contact and commitment we concluded that the introduction of a semester spokesperson and fixed meeting contribute to transparency of the current state and development of medical training. By contact and commitment all relevant parties of the construction of the medical curriculum are included which encourages community. The strong motivation and willingness of the semester spokespeople to take on responsibility for the overall process, despite the additional workload, was more clearly demonstrated in this study than had been anticipated. Possible reasons for their strong motivation and willingness were their active involvement in their own learning and that they perceived their learning environment as supportive and aiding. Furthermore, they also participated in the curriculum implementation as they were close to their teachers and fellow students. Moreover, the Community of Practice can promote education for all as all medical students have to manage the same tasks and challenges in their medical education. As a community they feel strengthened and empowered to meet these challenges. They support each other, e.g., in kind of student tutorials taught by peers. In this context, the social relationship between the students presents a relevant aspect for their learning success like Lave and Wenger have already reported (38). However, this only represented a small part of the student body. In the future, it would be interesting to interview different stakeholders, have follow-ups with former semester spokespeople or find out in what context the driving factors can be transferred to a larger cohort in order to exploit the potential and give more students access to these experiences as wonderfully mirrored by one of our student’s quotes: *“You have the feeling that you are being taken seriously, all the parts really are essential–it needs all of the parts to work properly. We are a group of people who want a good education for all of us” (Student G, 8th semester, 3rd semester in office).*

## Data Availability Statement

The original contributions presented in the study are included in the article/supplementary material, further inquiries can be directed to the corresponding author/s.

## Ethics Statement

The studies involving human participants were reviewed and approved by the Ethics Committee of Tübingen Medical Faculty (No. 058/2011A). The patients/participants provided their written informed consent to participate in this study.

## Author Contributions

FH was responsible for the design and conduction of the study, as well as acquisition, analysis, and interpretation of data, and drafted the first version of the manuscript. CK and TL were involved in data acquisition, analyses, and interpretation, and revised the manuscript critically. MH, ML-K, and SZ made substantial contributions to the study design and revised the manuscript critically. AHW supervised the study design and conduction as well as data collection and interpretation, and revised the manuscript critically. All authors approved the final version of the manuscript and agreed to be accountable for all aspects of the work.

## Conflict of Interest

The authors declare that the research was conducted in the absence of any commercial or financial relationships that could be construed as a potential conflict of interest.

## Publisher’s Note

All claims expressed in this article are solely those of the authors and do not necessarily represent those of their affiliated organizations, or those of the publisher, the editors and the reviewers. Any product that may be evaluated in this article, or claim that may be made by its manufacturer, is not guaranteed or endorsed by the publisher.

## Appendix

**TABLE 3:** Overview of codes.

Main category	Codes	Numbers of participants	Numbers of mentioning	Rank*
Role of spokesperson	Setting limits	7	11	1
	Responsibility	6	9	3
	Prioritisation	7	9	3
	Information gathering	6	9	3
	Mediator between students and faculty	6	7	5
Fixed meetings	Creating communication	6	10	2
	Transparency	7	10	2
	Structural limits	7	9	3
	Coming to a decision	3	3	8
Contact and commitment	Contacts and support offered	9	11	1
	Face-to-face meetings	8	11	1
	Continuous connection	8	10	2
	Objectivity of the spokesperson	6	9	3
	Working as a team	6	8	4
	Legitimation of one’s own role	5	6	6
	Exchanging information	5	6	6

**The rank is defined as followed: Rank 1 is highest, rank 8 is lowest.*
